# Antioxidant, antihyperglycemic, and antidiabetic activity of *Apis mellifera* bee tea

**DOI:** 10.1371/journal.pone.0197071

**Published:** 2018-06-05

**Authors:** Janielle da Silva Melo da Cunha, Tamaeh Monteiro Alfredo, Jéssica Maurino dos Santos, Valter Vieira Alves Junior, Luiza Antas Rabelo, Emerson Silva Lima, Ana Paula de Araújo Boleti, Carlos Alexandre Carollo, Edson Lucas dos Santos, Kely de Picoli Souza

**Affiliations:** 1 Binational Campus Oiapoque, Federal University of Amapá—UNIFAP, Amapá, Brazil; 2 Research Group on Biotechnology and Bioprospecting Applied to Metabolism, Federal University of Grande Dourados–UFGD, Dourados, Brazil; 3 School of Environmental and Biological Science, Federal University of Grande Dourados–UFGD, Dourados, Brazil; 4 Laboratory of Cardiovascular Reactivity–LRC, Nucleus of Metabolic Syndrome, Biological Sciences and Health Institute—ICBS, Federal University of Alagoas–UFAL, Alagoas, Brazil; 5 Faculty of Pharmaceutical Sciences, Federal University of Amazonas, Manaus, Brazil; 6 Laboratory of Natural Products am Mass Spectrometry, Federal University of Mato Grosso do Sul–UFMS, Mato Grosso do Sul, Brazil; Stellenbosch University, SOUTH AFRICA

## Abstract

Diabetes has emerged as one of the largest global epidemics; it is estimated that by 2035, there will be 592 million diabetic people in the world. Brazilian biodiversity and the knowledge of traditional peoples have contributed to the treatment of several diseases, including diabetes. *Apis mellifera* bee tea is used by indigenous Brazilians to treat diabetes, and this traditional knowledge needs to be recorded and studied.The objective of this study was to record the use and to evaluate the antioxidant, antihyperglycemic, and antidiabetic activity of *Apis mellifera* bee tea, which is used by the Guarani and Kaiowá indigenous people for the treatment of diabetes. Semi-structured interviews were performed with Guarani and Kaiowá ethnic indigenous people from the State of Mato Grosso do Sul, Brazil, seeking to identify the animal species used for medicinal purposes. For the experimental procedures, tea prepared with macerated *Apis mellifera* bees was used. In vitro assays were performed to evaluate antioxidant activity; direct free radical scavenging, protection against oxidative hemolysis, lipid peroxidation were evaluated in human erythrocytes and potential in inhibiting the formation of advanced glycation end products (AGEs). In vivo, normoglycemic Swiss male mice treated with *Apis mellifera* tea (AmT) were subjected to the oral glucose tolerance test and compared with control and metformin-treated groups. Diet-induced diabetic mice were treated for 21 days with AmT and evaluated for glycemia and malondialdehyde levels in the blood, liver, nervous system, and eyes. During interviews, the indigenous people described the use of Apis mellifera bee tea for the treatment of diabetes. In in vitro assays, AmT showed direct antioxidant activity and reduced oxidative hemolysis and malondialdehyde generation in human erythrocytes. The AmT inhibited the formation of AGEs by albumin-fructose pathways and methylglyoxal products. In vivo, after oral glucose overload, normoglycemic mice treated with AmT had reduced hyperglycemia at all times evaluated up to 180 min. AmT also reduced hyperglycemia and malondialdehyde levels in the blood, liver, nervous system, and eyes of diabetic mice to similar levels as those in metformin-treated mice and normoglycemic controls. In summary, Apis mellifera bee tea showed antioxidant, antihyperglycemic, and antidiabetic activity, which provides support for the therapeutic application of Guarani and Kaiowá indigenous knowledge.

## Introduction

In Brazil, several animal species are used for medicinal purposes in folk medicine and by indigenous communities [1], and much of this knowledge has not been scientifically described or proven [2]. The zootherapeutic knowledge of the Guarani and Kaiowá ethnic groups, who are located in the center-west region of Brazil, has not been sufficiently studied.

Thus, we chose to study the zootherapy used by the Guarani and Kaiowá ethnicities, who reported the use of *Apis mellifera* bee tea for the treatment of diabetes. Research on zootherapy is very important for cultural maintenance and to measure pharmacological effects [1,2,3].

Apiculture substances have been used since ancient times for many therapeutic purposes [4]. Examples include the use of honey in the treatment of wounds and burns [5,6], propolis as an antioxidant and antitumor agent [7,8], and bee venom for the reduction of complications arising from diabetes, which is an effect mediated by decreased human hemoglobin glycation [9]. In addition, some components isolated from bee venom, such as melittin, have anti-inflammatory and anti-carcinogenic effects [10,11], and tryptophan suppresses the elevation of blood glucose and preserves the insulin secretion from β-cells [12], and also considered a new marker associated with diabetes decreased risk [13].

In this context, several products derived from bees have been used in the prevention and treatment of diseases related to oxidative stress and diabetes [14,15,16]. The antioxidants are capable of directly enhancing the endogenous defense system [17] and modulating the enzymatic systems [18, 19] involved in reducing reactive species, especially reactive oxygen species (ROS). These activities ultimately prevent cell damage resulting from cell membrane lipid peroxidation [20], the oxidation of hormone receptors [21], and changes in the genetic material [22], which can lead to irreversible systemic damage such as nephropathy [23], retinopathy [24], and peripheral neuropathy [25]. These complications, in addition to their individual relevance, are aggravated in the context of diabetes [26, 27].

It is estimated that the global prevalence of diabetes will increase from 382 to 592 million people between 2013 and 2035 [28] because of factors such as aging, sedentary lifestyle, eating habits, and obesity. Obesity is associated with the increased production of ROS species [29, 30], and it is one of the main risk factors for the development of type 2 diabetes, which is characterized by insulin resistance and imbalanced glycemic homeostasis [31,32,33].

Thus, compounds with antioxidant properties may be a therapeutic alternative because they can reduce oxidative stress, hyperglycemia, and diabetes complications [26,34,35].

From this perspective, this is the first study that describes and evaluates the antioxidant, antihyperglycemic, and antidiabetic activity of *Apis mellifera* bee tea used by Guarani and Kaiowá indigenous people for the treatment of diabetes.

## Materials and methods

### Zootherapeutic knowledge

Semi-structured interviews (S1 Text) were conducted to record information on the species and animal resources used in the traditional medicine of the indigenous peoples of the Guarani and Kaiowá ethnic groups living in Mato Grosso do Sul, Brazil. Twenty (20) students of the Federal University of Grande Dourados (UFGD, Dourados, Brazil) belonging to the Guarani and Kaiowá ethnic groups consented to interviews (authorized by the Ethics Committee for research involving human subjects of UFGD under No. 1.858.827).

The Guarani and Kaiowá ethnic groups are distributed in 21 of the 79 municipalities of Mato Grosso do Sul; together they account for an estimated population of 42,000 and represent the second largest indigenous population of Brazil.

### Tea preparation

Bees of the species *Apis mellifera* were captured from their hives in the apiary of the School of Biological Sciences of the Federal University of Grande Dourados and immediately frozen to avoid metabolic changes. The tea was prepared according to information learned in interviews as follows: ten bees were macerated and placed in 10 mL of hot water (approximately 100°C), which corresponds to approximately 0.1 g of bees per mL of water, for 10 min. After this infusion period, the *Apis mellifera* tea (AmT) was filtered (Whatman filter paper, grade 40) and kept at 10°C in the refrigerator until use, which was no more than 48 h after preparation.

### HPLC

The AmT was analyzed in an analytical CLAE (Shimadzu), with two LC-20AD pumps, SIL-20A auto-injector, SPD-M20A diode array detector (DAD), CBM-20A controller and CTO-20A oven. The chromatograph was also coupled to a microTOFIII mass spectrometer (Bruker Daltonics) with electrospray ionization source and quadrupole time-of-flight analyzer (Q-TOF). The chromatographic column used was Kinetex C-18 (150 x 2.1 mm, Phenomenex), coupled to a pre-column with the same material. The mobile phase was composed by acetonitrile (B) and deionized water (A), both containing 1% of formic acid (v/v) under a flow rate of 0.3 ml/min and the oven temperature was 50 oC. The gradient method applied was: 0–2 min. 3% B (isocratic), 2–25 min. 3 to 25% B, 25–40 min. 25 to 80% B and 40–43 min. to 80% B (isocratic). Followed by washing and reconditioning the column. The molecular formula of each compound was determined based on the error of up to 5 ppm and mSigma below 30.

### ABTS free radical scavenging activity

The antioxidant activity was determined *in vitro* by studying the scavenging of 2,2 'azino-bis (3-ethylbenzthiazoline-6-sulfonic acid) (ABTS) free radicals, as described previously by Larrauri et al., [17]. For this purpose, AmT was used at concentrations of 5, 50, 100, 500, and 1,000 μg/mL. A total of 20 μL of each AmT concentration was mixed with 1980 μL of ABTS. Ascorbic acid and butyl hydroxytoluene (BHT) standards were prepared at the same concentrations used for AmT, and three independent experiments were performed in duplicate. After 6 min, the absorbance at 734 nm was recorded with a UV/VIS spectrophotometer (PG instruments, Ltd.). The percentage of ABTS radical inhibition was calculated according to Eq 1:

Scavengingactivity(%)=(1−Abssample/Abscontrol)x100 (1)

### DPPH free radical scavenging activity

Free radical-scavenger activity was determined by the 2,2-diphenyl-1-picrylhydrazyl (DPPH) assay, as described previously by Gupta and Gupta [36], with some modifications. The antiradical activity of the extracts was evaluated using a dilution series, which involved the mixing of 1.8 mL of DPPH solution (0.11 mM DPPH in 80% ethanol) with 0.2 mL of AmT (5–1000 μg/mL). After 30 min, the remaining DPPH radicals were quantified by absorption at 517 nm. The absorbance of each concentration of the AmT was subtracted from absorbance of the samples with DPPH solution. Ascorbic acid and butylated hydroxytoluene (BHT) were used as reference antioxidants. The tests were performed in duplicate in three independent experiments. DPPH solution without the tested sample was used as a control. The percentage inhibition was calculated from the control with the following Eq 2:

Scavengingactivity(%)=(1−Abssample/Abscontrol)x100 (2)

### Oxidative hemolysis induced by AAPH

The experimental procedure was submitted to and approved by the Research Ethics Committee involving human beings of the UFGD (authorization No. 1.627.746). The ability of AmT to protect against oxidative hemolysis was evaluated by assessing hemolysis induced by the oxidizing agent 2,2'-azobis (2-methylpropionamidine) dihydrochloride (AAPH) [37]. Fifteen milliliters of human peripheral blood from a single healthy adult individual was collected. The blood was centrifuged at 2,000 rpm, and the plasma and the leukocyte layer were discarded. The erythrocytes were washed three times in 0.9% NaCl, and after washing, a 10% erythrocyte suspension in 0.9% NaCl was prepared. Subsequently, the material was incubated at 37C for 30 min in test tubes in the presence of different concentrations of ascorbic acid or AmT (50, 100, 500, 1000 μg/ml). Subsequently, 0.9% NaCl or 50 mM AAPH was added to evaluate the hemolytic capacity and the inhibition of oxidative hemolysis, respectively. The samples were kept at 37°C for 240 min with periodic stirring. Three independent experiments were performed in duplicate. The percentage of hemolysis was determined by measuring the absorbance at 540 nm and using Eq 3:

Hemolysis(%)=(Abssample/Abstotalhemolysis)x100 (3)

### Measurement of MDA in human erythrocytes

Lipid peroxidation assays were performed with 10% erythrocyte suspensions. The erythrocytes were incubated at 37°C for 30 min in the presence of different concentrations of ascorbic acid or AmT (50, 100, 500, 1000 μg/ml). Thereafter, 50 mM AAPH was added to the erythrocyte solution and incubated at 37°C for 4 h with periodic stirring. After this period of time, the samples were centrifuged at 2,000 rpm, and 500-μL aliquots of the supernatant were transferred to tubes containing 1 mL of 10 nmol thiobarbituric acid (TBA). As a standard, 500 μL of 20 μM malondialdehyde (MDA) solution was added to 1 mL of TBA. Samples were incubated at 96°C for 45 min. Then, 4 mL of n-butyl alcohol was added and centrifuged at 2,000 rpm. The absorbance of the supernatants was measured at 532 nm. Two independent experiments were performed in triplicate. The concentration of MDA in the samples is expressed in nmol/mL and was obtained from Eq 4:

MDA=Abssamplex(20x220.32/Absstandard) (4)

### Oral glucose tolerance test in normoglycemic mice

All experimental animal procedures were submitted to and approved by the UFGD Ethics Committee for Animal Use (authorization No. 25.2016) and performed according to the standards of the National Council for the Control of Animal Experimentation (CONCEA).

The oral glucose tolerance test (OGTT) was performed after 12 hours of fasting in 15 normoglycemic *Swiss* adult male mice weighing between 55 and 60 g. The mice were given water, metformin (100 mg/kg body weight), or AmT (200 mg/kg body weight), respectively forming the following groups: (1) Control, (2) Metformin, and (3) AmT. After 30 min of treatment, the mice received glucose overload by gavage (2 g/kg body weight). At times 0, 30, 60, 90, 120, and 180 min after the administration of glucose, the glycemia of the mice was measured using caudal venous blood [38] using the Accu-chek Active (Roche) glucose meter and specific glucose test strips.

### Measurement of glycemia and tissue MDA levels in diabetic mice

Approximately 60-day-old normoglycemic *Swiss* male mice were maintained under controlled temperature (22 ± 2°C) on a 12-hour light, 12-hour dark cycle with free access to food and water.

The mice were fed for 120 days with a control diet (standard Labina feed for rodents) or a high-calorie diet for the induction of hyperglycemia [32]. After this period, blood glucose levels were measured using the Accu-chek Active (Roche®) device and specific glucose test strips. Hyperglycemic mice (glycemia ≥ 200 mg/dL) fed a high-calorie diet were divided randomly (n = 5 per group) and treated for 21 days with water (D-Control Group), 100 mg/kg body weight metformin (D-Metformin Group), or 1 g/mL body weight AmT (D-AmT Group). Normoglycemic mice fed a control diet and water formed the ND-Control Group (n = 5).

At the end of the treatment, the blood glucose level of the mice was measured again. The mice were then anesthetized and euthanized. Arterial blood was collected by cardiac puncture, and the liver, nervous system, and eyes were collected for the measurement of MDA by a method adapted from [39]. Arterial blood samples and tissues were homogenized with 1.15% KCl (potassium chloride) and centrifuged at 3,000 rpm for 10 min. Then, 500 μL of the sample supernatant or 500 μL of the 20 μM MDA standard was added to 1 mL of 10% trichloroacetic acid (TCA) and 1 mL of 10 nmol thiobarbituric acid (TBA) and incubated at 96°C for 45 min. After cooling for 15 min in an ice bath, 3 ml of n-butyl alcohol was added, and the mixture was vortexed and centrifuged at 3,000 rpm for 5 min. Subsequently, the absorbance of 2 mL of the supernatant was measured at 532 nm on a UV/VIS spectrophotometer. The concentration of MDA in the samples is expressed in nmol/mL and was obtained from Eq (5):

MDA=Abssamplex(20x220.32/Absstandard) (5)

#### Biometric parameters of diabetic mice

Body weight (g), water consumption (mL), and feed (g) were evaluated three times per week during the 21 days of treatment. In addition, different deposits of white adipose tissue (epididymal, subcutaneous, mesenteric, and retroperitoneal) were removed and weighed after euthanasia.

### Glycation inhibition assay

The potential in inhibiting glycation was performed considering the fructose and methylglyoxal pathway as described by Kiho et al., [40]. Bovine serum albumin 8 mg/mL, 0.1mM fructose and 30 mM methylglyoxal were prepared in phosphate buffer 0.2 M, pH 7.4, containing 3 mM sodium azide, as an antimicrobial agent. To 30 μl AmT (1–1000 μg/ml) was mixed with 135 μl of bovine serum albumin and 135 μl of fructose or 135 μl of methylglyoxal. The mixture reaction was incubated at 37ºC for 48 h or 72 h (under sterile conditions in the dark), for inhibition of glycation fructose or glyoxal pathway, respectively. After this period, each sample was examined for the development of fluorescence using a microplate reader DTX 800, Beckman (λex 330 nm and λex 420 nm) against a blank. Quercetin (1–1000 μg/ml) was used as standard. The control consisted of mixing 30 μl of 80% ethanol with 135 μl of bovine serum albumin and 135 μl of fructose or 135 μl methylglyoxal. Three independent experiments were carried out in triplicate. The percentage of inhibition of glycation was obtained by the Eq (6) and IC_50_ values were obtained by non-linear regressions of the concentration-response curve.

Inhibitionglycation(%)=[1−(Fluorescence/Fluorescence)]x100 (6)

### Statistical analysis

Results are expressed as the means ± standard error of the mean. Analysis of variance (ANOVA) followed by post-test Student-Newman-Keuls analysis was used for multiple comparisons of results, and Student’s t test was used to compare the results of both groups using the Prism 5 GraphPad software. The level of significance was P <0.05.

## Results

### Zootherapeutic knowledge

In the interviews of twenty Guarani and Kaiowá indigenous people aged between 20 and 40 years concerning the animal species used in traditional medicine, 30% described the use of *Apis mellifera* bee tea for the treatment of diabetes. This use was reported by individuals aged between 30 and 40 years.

### HPLC

The AmT was filtered and analyzed in HPLC-DAD-MS/MS in the positive and negative ionization modes, we observed a significant ionization only in the positive ionization mode (S1 Fig) which was used to characterize Tryptophan derivative, as well as 16 other compounds that still need to be identified (S1 Table).

### Antioxidant activity

#### ABTS and DPPH free radical scavenging

AmT showed excellent direct antioxidant activity with an IC_50_ value similar to that of BHT and higher than that of ascorbic acid (Table 1).

**Table 1:** ABTS and DPPH free radical scavenging activity of *Apis mellifera* bee tea (AmT) and the standard antioxidants ascorbic acid (AA) and BHT.

Sample	ABTS			DPPH		
	IC_50_	Maximum inhibition		IC_50_	Maximum inhibition	
	μg/mL	%	μg/mL	μg/mL	%	μg/mL
AA	3.39 ± 0.02	97.30 ± 0.14	10	4.55 ± 0.05	94.77 ± 0.11	50
BHT	13.12 ± 0.13	95.79 ± 0.26	50	21.21 ± 0.8	86.77 ± 0.18	500
AmT	15.63 ± 0.06	95.33 ± 0.68	100	33.74 ± 0.8	87.25 ± 0.44	500

IC_50_, concentration required to capture 50% of ABTS and 50% of DPPH free radicals from the reaction. Values are expressed as the means ± SEM.

#### AAPH-Induced oxidative hemolysis

**Fig 1:**
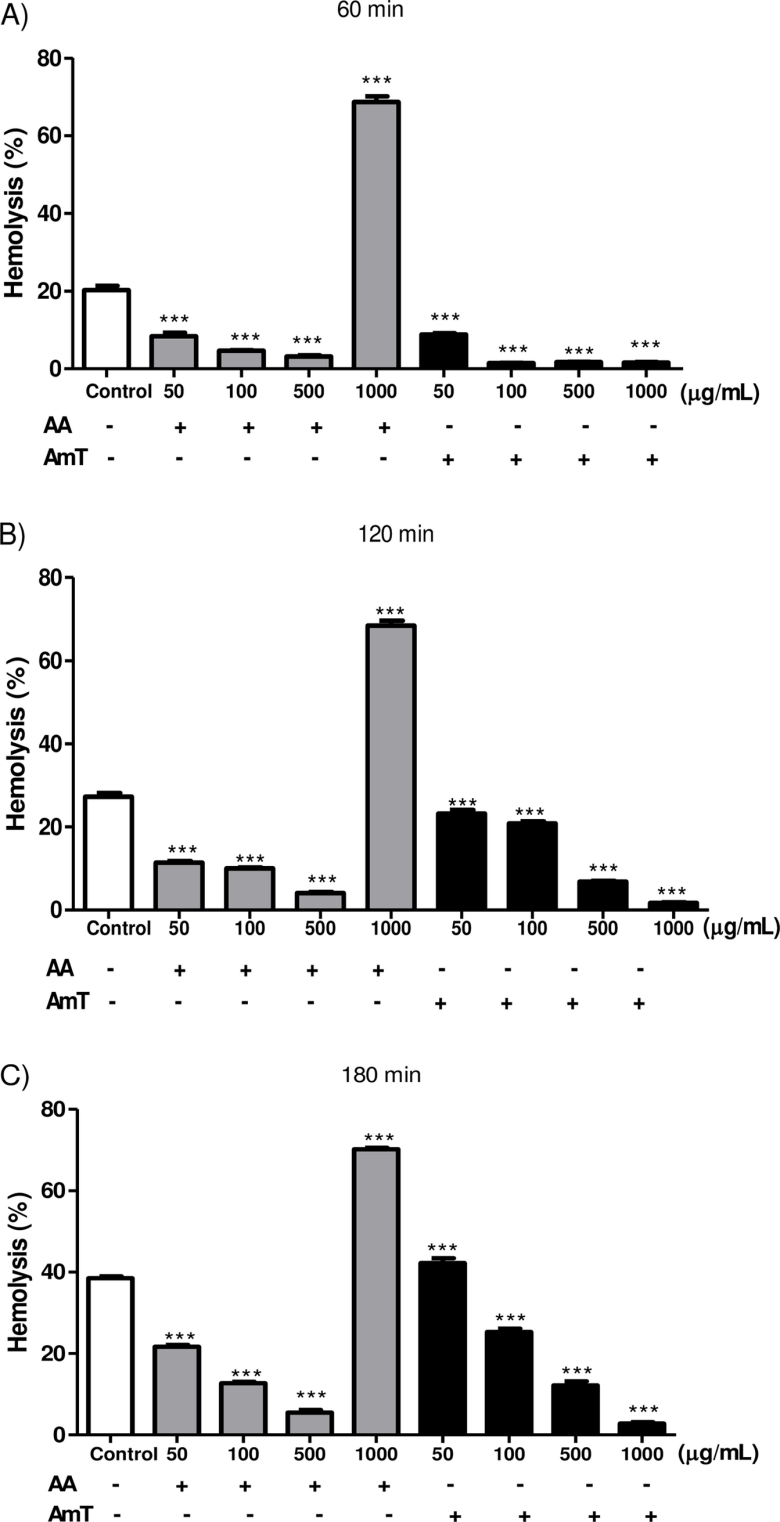
Oxidative hemolysis induced by AAPH. Human erythrocytes incubated with different concentrations (50–1,000 μg/mL) of ascorbic acid (AA) and *Apis mellifera* bee tea (AmT) (A) 60, (B) 120, and (C) 180 min after the addition of AAPH. Values are presented as the means ± SEM. * P<0.05; ** P<0.01; and *** P<0.001 versus Control sample.

The antioxidant activity of AmT was also confirmed by the scavenging of free radicals generated by AAPH thermolysis in the human erythrocyte assay. AmT efficiently reduced erythrocyte oxidative hemolysis in a dose- and time-dependent manner, similar to all concentrations of ascorbic acid (Fig 1) except for 1,000 μg/mL, a dose at which ascorbic acid behaved as a pro-oxidant substance.

#### Measurement of MDA in human erythrocytes

**Fig 2:**
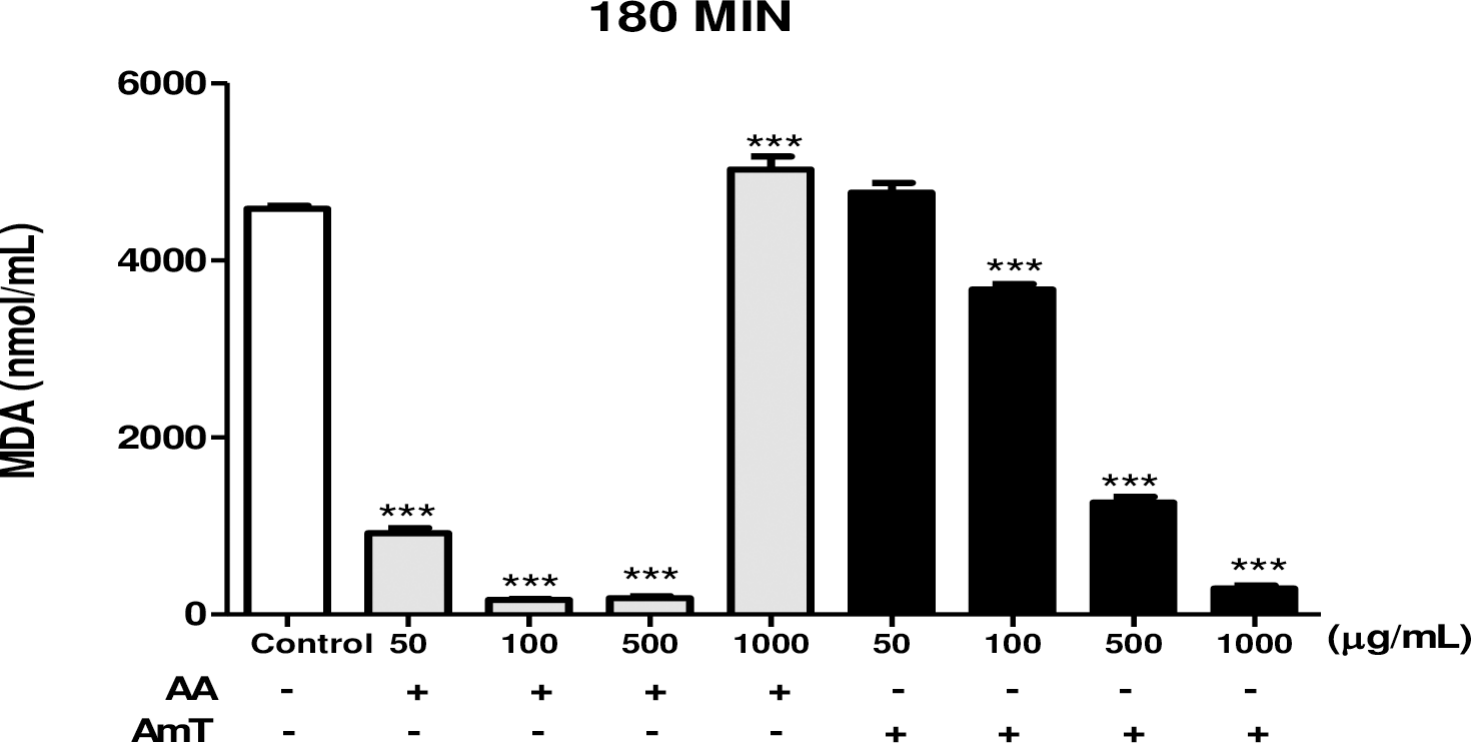
Measurement of MDA in human erythrocytes. Malondialdehyde (MDA) concentration 180 min after the addition of the oxidizing agent (AAPH) in erythrocytes incubated with different concentrations (50–1,000 mg/mL) of ascorbic acid (AA) or *Apis mellifera* bee tea (AmT). Values are presented as the means ± SEM. *P<0.05; **P<0.01; and ***P<0.001 versus Control sample.

Once protection against oxidative hemolysis was confirmed, its correlation with the reduction of lipid peroxidation was investigated by evaluating the malondialdehyde (MDA) marker in erythrocytes induced with AAPH. Fig 2 indicates that erythrocytes incubated with concentrations greater than 100 μg/mL AmT demonstrated reduced generation of MDA, similar to the observations in erythrocytes incubated with ascorbic acid, except at the highest evaluated concentration of 1,000 μg/mL, at which the ascorbic acid demonstrated pro-oxidant activity.

### Antihyperglycemic activity

#### Oral glucose tolerance test in normoglycemic mice

**Fig 3:**
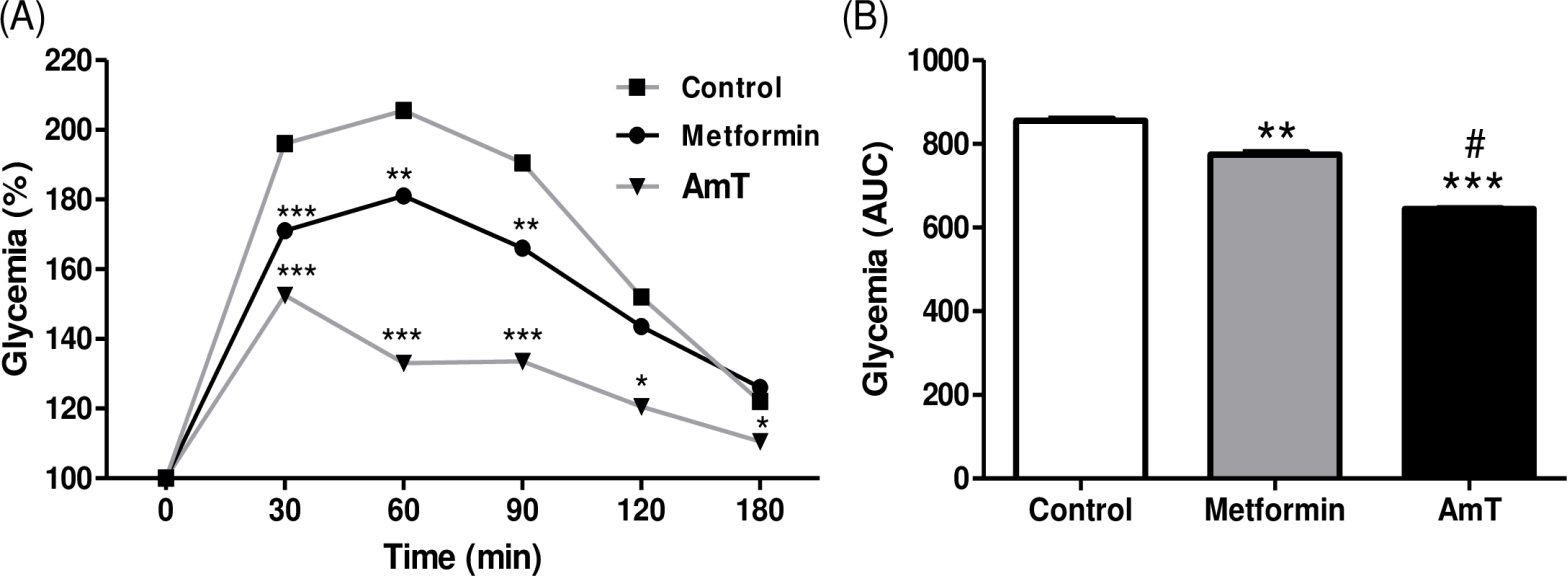
Oral glucose tolerance test in normoglycemic mice. (A) Variation and (B) area under the glycemia curve at 0, 30, 60, and 180 min for mice treated with water, metformin, and AmT after glucose overload. *P<0.05; **P<0.01; and ***P<0.001 versus Control group. ^#^P <0.001 vs. Metformin group.

After oral glucose overload, the normoglycemic mice treated with AmT showed a reduced hyperglycemia peak after 30 min and lower blood glucose values at all times evaluated up to 180 min compared to the control mice (Fig 3A). This reduction in serum hyperglycemia can also be observed in the area under the curve, which shows that AmT is more efficient compared with both control and metformin treatment (Fig 3B).

### Antidiabetic activity

#### Glycemia of diabetic mice

**Fig 4:**
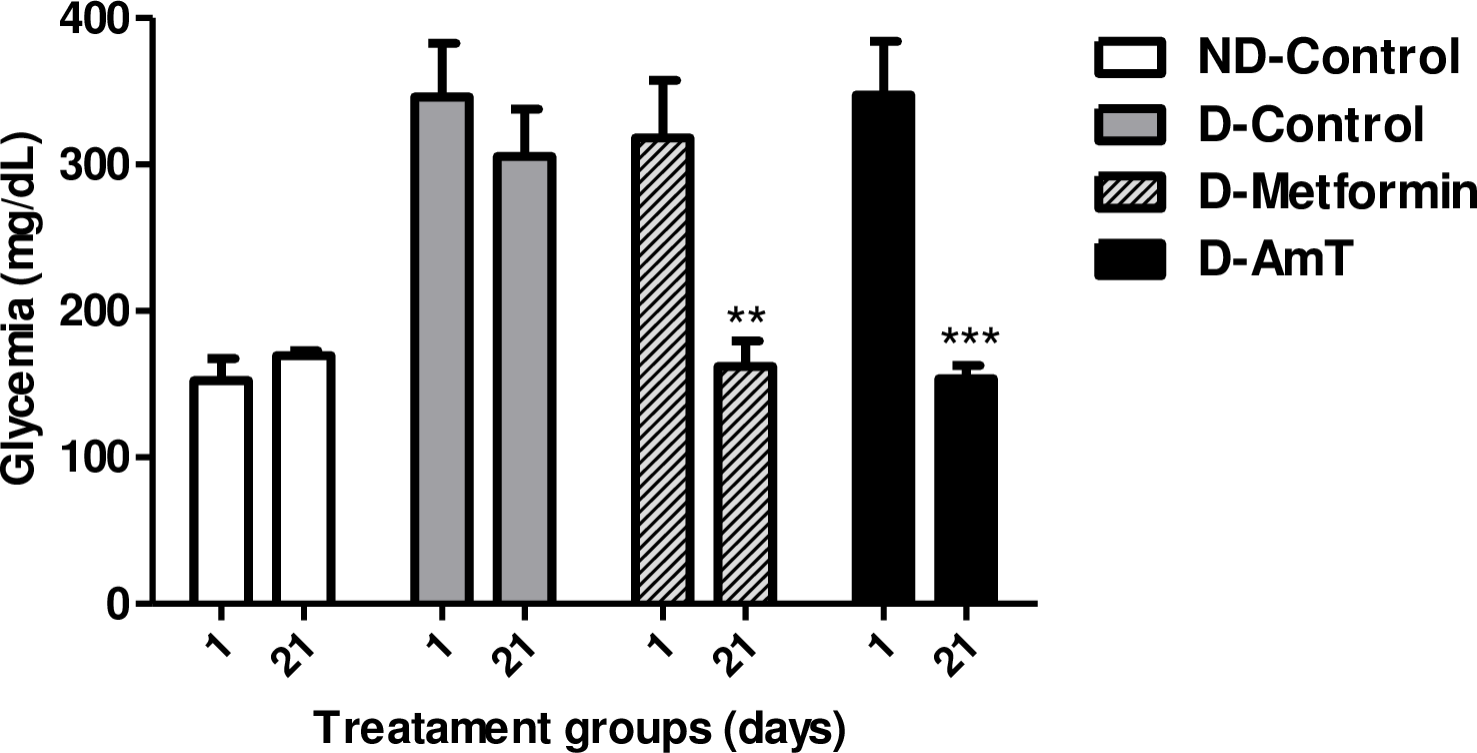
Glycemia of normoglycemic and high-calorie diet-induced diabetic mice treated for 21 days. Glycemia in mice from the ND-Control (normoglycemic + water), D-Control (diabetic + water), D-Metformin (diabetic + metformin), and D-AmT (diabetic + AmT) groups on days 1 and 21 of treatment. Values were compared between days 1 and 21 for each experimental group. **P<0.01 and ***P<0.001 versus day 1 of the respective group.

The evaluation of glycemia in high-calorie diet-induced diabetic mice on the first (1st) and last (21st) day of treatment demonstrated the antidiabetic activity of AmT. Fig 4 demonstrates that AmT reduced the hyperglycemia of diabetic mice similarly to treatment with metformin and to normoglycemic controls.

#### Biometric parameters of diabetic mice

The biometric parameters evaluated during the treatment are presented in Table 2. The diabetic mice presented reduced body weights compared to normoglycemic mice, although no variation in feed consumption was observed between groups. Water intake of diabetic mice was approximately 3-fold higher than that of normoglycemic mice. Treatment with AmT reduced water intake to similar levels as the normoglycemic control and metformin groups. In addition, a reduction of subcutaneous WAT is seen in diabetic mice treated with water compared to diabetic mice treated with metformin or AmT or to normoglycemic mice. The other WAT deposits evaluated were similar among the groups investigated.

**Table 2:** Biometric parameters and consumption of feed and water of non-diabetic (ND) mice treated with water (D-Control), metformin (D-Metformin), and *Apis mellifera* bee tea (D-AmT) for 21 days.

Parameters	ND-Control	D-Control	D-Metformin	D-AmT
Δ body weight _(%)_	5.9 ± 0.2^a^	-13.7 ± 0.4^b^	-9.8 ± 0.3^c^	-13.8 ± 0.3^b^
Feed consumption _(g/day/animal)_	4.2 ± 0.5^a^	3.3 ± 0,4^a^	3.9 ± 0,7^a^	3.9 ± 0,4^a^
Water intake _(mL/day/animal)_	2.7 ± 0.3^a^	8.3 ± 0.7^b^	2.1 ± 0.4^a^	1.8 ± 0.4ª
Subcutaneous WAT _(g/100 g of BW)_	0.29 ± 0.07^a^	0.08 ± 0.02^b^	0.18 ± 0.05^a^	0.23 ± 0.06^a^
Mesenteric WAT _(g/100 g of BW)_	0.68 ± 0.15^a^	1.12 ± 0.27^a^	0.96± 0.26^a^	1.23 ± 0.31^a^
Epididymal WAT _(g/100 g of BW)_	2.99 ± 0.30^a^	2.09 ± 0.41^a^	1.63 ± 0.49^a^	1.97 ± 0.26^a^
Retroperitoneal WAT _(g/100 g of BW)_	0.75 ± 0.11^a^	0.71 ± 0.08^a^	0.78 ± 0.20^a^	0.98 ± 0.22^a^

Δ, body weight variation between the beginning and end of the 21-day treatment expressed in %; WAT, white adipose tissue. Different letters indicate P<0.05 between groups.

#### Tissue MDA levels in diabetic mice

**Fig 5:**
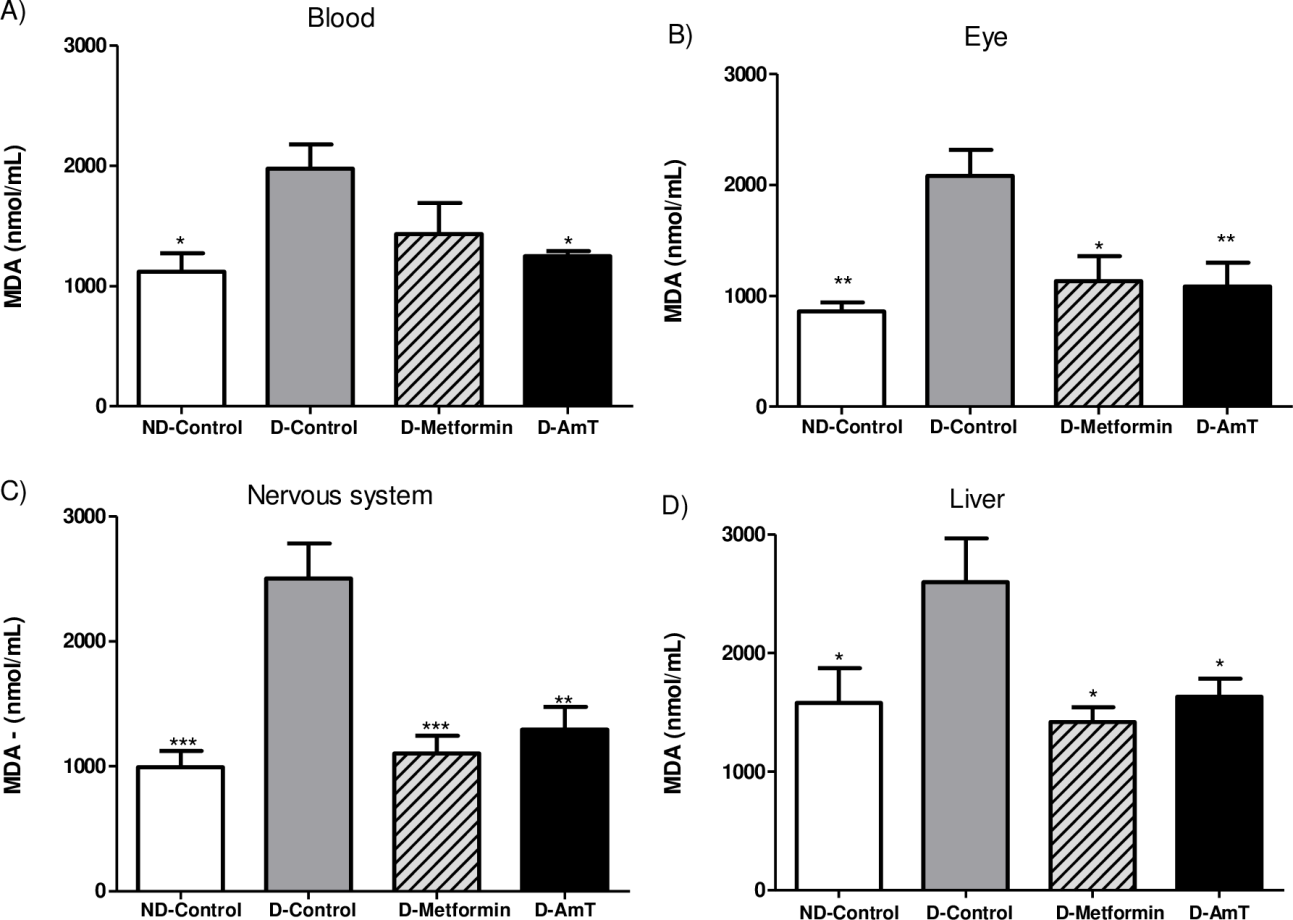
Malondialdehyde concentration (MDA) in the arterial blood, liver, nervous system, and eyes of normoglycemic and diabetic mice. Groups include ND-Control (normoglycemic + water), D-Control (diabetic + water), D-Metformin (diabetic + metformin), and D-AmT (diabetic + AmT) after 21 days of treatment. Data are presented as the means ± SEM. * P<0.05; ** P<0.01; and ***P <0.001 versus D-Control group.

Compared to normoglycemic mice, diabetic mice treated with water (D-Control) presented increased levels of MDA in the arterial blood (76%), liver (64%), nervous system (152%), and eyes (142%) (Fig 5). AmT-treated diabetic mice showed decreased levels of MDA in the blood (37%), liver (37%), nervous system (48%), and eyes (48%) compared to the D-Control group and similar levels as those of the metformin and normoglycemic control groups (Fig 5). No differences were observed in the relative mass of the organs between the evaluated groups.

#### Glycation inhibition assay

The potential in inhibiting the formation of advanced glycation end products (AGEs) are presented in Table 3. The AmT was able to inhibit 53.23% of glycation by the methylglyoxal pathway and 98.73% by the fructose pathway.

**Table 3:** Glycation inhibition assay.

Sample	Methylglyoxal			Fructose		
	IC_50_	inhibition		IC_50_	inhibition	
	μg/mL	%	μg/mL	μg/mL	%	μg/mL
**Quercetin**	35.57 ± 0.85	80.13 ± 0.34	100	0.31 ± 0.02	99.23 ± 0.84	100
**AmT**	932.63 ± 0.13	53.23 ± 1.54	1000	111.67 ± 3.51	98.73 ± 2.82	1000

The potential for inhibition of glycation by the methylglyoxal and fructose pathways for AmT and Quercetin Control. IC_50_ values were obtained by non-linear regressions of the concentration-response curve.

## Discussion

Traditional indigenous knowledge about zootherapy is the result of centuries of accumulated experiences and cultural practices. However, with increased globalization and interculturality, this knowledge, which remains poorly recorded, is being lost over time. Records and scientific research are needed so that experiences accumulated over centuries will not be lost, thus depriving future generations and other communities of this knowledge. Research surveying zootherapies used in Brazil is a recent development [2,41,42], and no study of the knowledge of the Guarani and Kaiowá ethnicities has been performed. Thus, this study contributes to the maintenance of this information and to cultural revitalization by recording and evaluating the use of *Apis mellifera* bee tea for the treatment of diabetes.

In the characterization of chemical compounds, compound 1 showed a UV compatible with Tryptophan and an m/z 188.0706 compatible with the tryptophan skeleton without the ammonium group, as well as 16 other compounds that still need to be identified. The ability to tryptophan of reducing the elevation of blood glucose and improving insulin secretion from β-cells [12] may have contributed for glycemic normalization in diabetic mice treated with AmT. The presence of tryptophan in the blood has been considered a marker of lower risk of diabetes development [13]. For the other compounds we observed two series of compounds, compounds 3 and 4 with the formulas C_15_H_22_O_9_ and C_16_H_24_O_10_, respectively, both without significant UV absorption and without MS/MS fragments. The search for similar compounds in the literature did not return results. The second series as formed by the compounds number 2, 5–17 which have two nitrogen in their composition with increasing carbonic chain length (C_10_ to C_20_), in addition to 1 or 2 oxygen, no one of these compounds also absorbed in the UV spectrum. Again the search for compatible compounds in literature did not return positive results. Several authors demonstrate the presence of waxes in the body composition of bees and other insects, but these do not present nitrogen in their composition [43, 44]. We believe that both classes of compounds could be new in the literature; unfortunately a positive characterization will require other work with this focus.

Oxidative stress promotes insulin secretion defects, decreases insulin sensitivity in peripheral tissues, and causes other complications associated with diabetes, and oxidative stress control is helpful in diabetes treatment [45]. Antioxidant activity is important in biological systems exposed to excess reactive species. For example, muscle cells cultured in the presence of an ROS inducer show reduced glucose uptake that is overcome upon treatment with antioxidant substances [45, 46].

In the present study, AmT showed potential antioxidant, antihyperglycemic, and antidiabetic activities. The antioxidant activity of AmT was demonstrated *in vitro* by the direct scavenging of ABTS and DPPH reactive species, the inhibition of lipid peroxidation induced by AAPH, and the reduction of oxidative hemolysis and malondialdehyde production in human erythrocytes. The peroxidation of polyunsaturated fatty acids in the cell membrane, which is induced by excess free radicals [47], causes damage and cell death [48] and the release of malondialdehyde (MDA), a byproduct derivative of lipid peroxidation [49]. Our results suggest a synergy between compounds with antioxidant properties obtained from macerated whole bees compared to synthetic antioxidants, ascorbic acid, and BHT. Recently, [50] observed that *Apis mellifera iberiensis* bee venom had antioxidant activity, but they could not attribute this activity to the major compounds, which were identified as melittin, phospholipase A2, and apamine.

The antihyperglycemic activity of AmT was indicated *in vivo* by the control of postprandial hyperglycemia in normoglycemic mice subjected to glucose overload, and this activity was similar to that of metformin. Although the main cellular damage related to diabetes is caused by prolonged exposure to hyperglycemia, strategies to control postprandial hyperglycemia peaks have been associated with reduced vascular damage in diabetic patients [51, 52]. Considering the short time between the administration of AmT and improved glycemic control, it is possible to infer that rapidly acting mechanisms are being activated, such as GLUT4 translocation in skeletal muscle tissue and white adipose tissue [53] or the activation of kinases, which is important in processes related to insulin sensitivity and glucose uptake [54], but these mechanisms still need to be investigated. Additionally, [55], reported that the administration of bee venom (apitoxin) in alloxan-induced diabetic rats increased serum insulin secretion and reduced blood glucose levels. However, melittin, the main constituent of apitoxin, improves insulin sensitivity through the activation of phospholipase A2 in diabetic mice induced by diet [56]. Both apitoxin and melittin may have contributed to the glycemic control observed in this study, although insulin resistance is the predominant mechanism that triggers diet-induced diabetes [57, 58, 59], which was the model used in this study.

In addition, in diabetic mice treated with AmT, the reduction of glycemic parameters to levels similar to metformin and normoglycemic controls and the reduction of lipid peroxidation were observed in the different tissues and organs evaluated.

The involvement of oxidative stress in the etiology and progression of diabetes has created opportunities for the development of antidiabetic therapies linked to the blockade of oxidative pathways with the use of antioxidant substances [40, 60]. In diabetes, hyperglycemia intensifies oxidative stress through mechanisms involving increased aldose reductase enzyme activity, protein kinases [61], and formation of advanced glycation end products [62]. Protein glycation occurs commonly in non-enzymatic reactions, called the Maillard reaction, occurring between free amino groups of proteins and carbonyl groups of reducing sugars such as glucose, fructose, pentoses, galactose, mannose and xylulose, forming a compound unstable, the Schiff base, which undergoes a rearrangement to a more stable product known as Amadori product [63]. The Amadori product degrades to a variety of reactive dicarbonyl compounds, such as methylglyoxal and deoxyglucosones, by dehydration, oxidation and other chemical reactions [64]. In the formation of AGEs, reactive oxygen species can be generated and increase oxidative stress leading to structural and functional damage of macromolecules [40, 63, 64]. The ability of AmT to inhibit the formation of AGEs by fructose pathways was nine times more significant than the inhibition of the reaction of methylglyoxal products, increasing the possibility that this is one of the mechanisms of action of the AmT, contributing to the improvement of glycemic control and complications of diabetes. Metformin is effective in the control of diabetes because it has antioxidant and antihyperglycemic activities that are mediated by different mechanisms [65, 66].

The periodic evaluation of lipid peroxidation products in diabetes is recommended because their detection and early treatment reduce the diabetic complications, such as endothelial dysfunction, neuropathy, and retinopathy, that are mediated by excess reactive species reaching different tissues [67, 68]. The protective effect of AmT was indicated by the reduction of lipid peroxidation in the organs and tissues of diabetic mice; these mice presented levels of MDA similar to normoglycemic animals at the end of treatment. This effect was also observed in diabetic rats treated with combinations of probiotics and vitamin C [69]. In addition, parameters that demonstrate improvement in diabetes, such as the normalization of water intake and the maintenance of the deposition of subcutaneous white adipose tissue [33, 70], were observed in this study.

## Conclusions

*Apis mellifera* bee tea, a traditional diabetes treatment used by the indigenous Guarani and Kaiowá people groups, in this study showed potential antioxidant, and was efficient in controlled the postprandial hyperglycemia of normoglycemic mice, and normalized glycemia in diabetic mice. These data are important because they contribute to the recording of traditional zootherapeutic knowledge used for the treatment of diabetes.

## Supporting information

S1 Text Semi-structured interview model applied to register traditional knowledge. (DOCX)

S1 Fig Chromatography profile of the *Apis mellifera* Tea (AmT). (TIF)

S1 Table Compounds characterized by HPLC-DAD-MS/MS in *Apis mellifera* Tea (AmT). (DOCX)

S1 File Checklist. (PDF)
