# Supplementary material for: Antioxidant, antihyperglycemic, and antidiabetic activity of Apis mellifera bee tea
Source: PLoS One. 2018 Jun 5;13(6):e0197071. doi: 10.1371/journal.pone.0197071 (PMC5988306; doi:10.1371/journal.pone.0197071)
Supplement: S1 Text — (DOCX) [file pone.0197071.s001.docx]

**S1 Text.** Semi-structured interview model applied to register traditional knowledge

1- Years old ____________________

2- Sex: woman ( ) man( )

3. Occupation: ( ) student, ( ) technician ( ) teacher

4. Do you belong to any traditional community?

( ) not ( ) yes. What? ( ) quilombola ( ) from Camp ( ) indigenous ( ) other ___________________________________________________.

5. Are you aware of any natural remedy produced with parts of animals?

( ) not ( ) yes. Complete the following table.

| Vernacular name | Indication | Part used | Mode of preparation |
| --- | --- | --- | --- |
|  |  |  |  |
|  |  |  |  |
|  |  |  |  |
|  |  |  |  |
|  |  |  |  |
|  |  |  |  |
|  |  |  |  |
|  |  |  |  |
