# Supplementary figures and images for: Antioxidant, antihyperglycemic, and antidiabetic activity of Apis mellifera bee tea
Source: PLoS One. 2018 Jun 5;13(6):e0197071. doi: 10.1371/journal.pone.0197071 (PMC5988306; doi:10.1371/journal.pone.0197071)

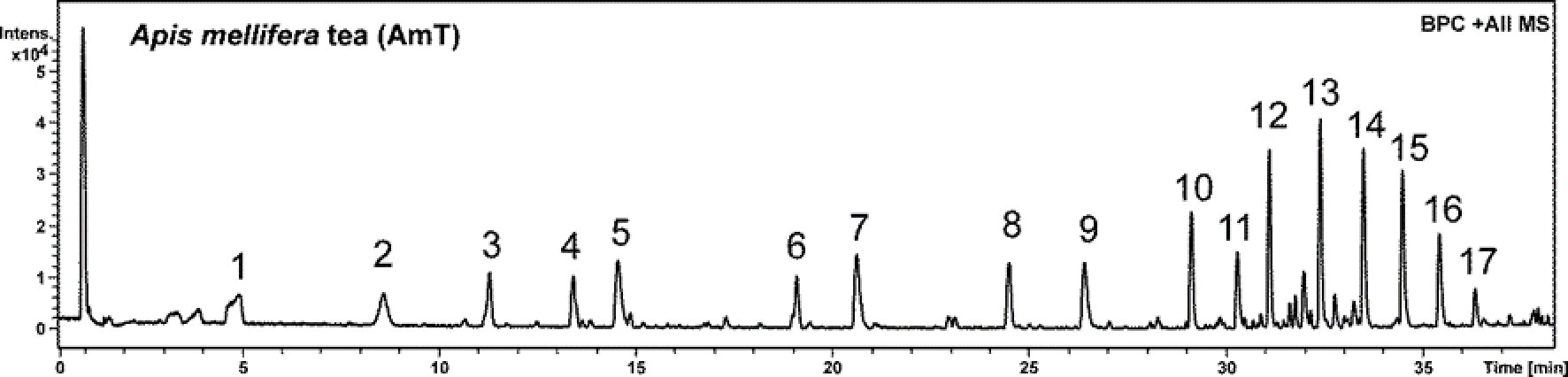

Supplement: S1 Fig — (TIF) [file pone.0197071.s002.tif]
