# Supplementary material for: Antioxidant, antihyperglycemic, and antidiabetic activity of Apis mellifera bee tea
Source: PLoS One. 2018 Jun 5;13(6):e0197071. doi: 10.1371/journal.pone.0197071 (PMC5988306; doi:10.1371/journal.pone.0197071)
Supplement: S1 Table — (DOCX) [file pone.0197071.s003.docx]

**S1 Table. Compounds characterized by HPLC-DAD-MS/MS in *Apis melifera* Tea (AmT).**

| ID | Compound | Retention Time | [M+H]^+^ (*m/z*) | Error (ppm) | Molecular formula |
| --- | --- | --- | --- | --- | --- |
| 1 | Tryptophan derivative | 5.0 | 188.0706 | 0.9 | C_11_H_9_NO_2_ |
| 2 | Unknown | 8.7 | 181.1335 | 2.0 | C_10_H_16_N_2_O |
| 3 | Unknown | 11.3 | 347.1337 | 3.1 | C_15_H_22_O_9_ |
| 4 | Unknown | 13.5 | 277.1442 | 0.0 | C_16_H_24_O_10_ |
| 5 | Unknown | 14.6 | 195.1499 | 3.4 | C_11_H_18_N_2_O |
| 6 | Unknown | 19.2 | 225.1598 | 4.2 | C_12_H_20_N_2_O_2_ |
| 7 | Unknown | 20.7 | 209.1648 | 4.4 | C_12_H_20_N_2_O |
| 8 | Unknown | 24.5 | 239.1754 | 0.7 | C_13_H_22_N_2_O_2_ |
| 9 | Unknown | 26.4 | 223.1805 | 2.3 | C_13_H_22_N_2_O |
| 10 | Unknown | 29.2 | 253.1919 | 3.3 | C_14_H_24_N_2_O_2_ |
| 11 | Unknown | 30.3 | 237.1981 | 1.2 | C_14_H_24_N_2_O |
| 12 | Unknown | 31.1 | 267.2069 | 0.8 | C_15_H_26_N_2_O_2_ |
| 13 | Unknown | 32.4 | 281.2230 | 2.3 | C_16_H_28_N_2_O_2_ |
| 14 | Unknown | 33.5 | 295.2385 | 1.5 | C_17_H_30_N_2_O_2_ |
| 15 | Unknown | 34.5 | 309.2548 | 3.7 | C_18_H_32_N_2_O_2_ |
| 16 | Unknown | 35.5 | 323.2693 | 4.9 | C_19_H_34_N_2_O_2_ |
| 17 | Unknown | 36.4 | 337.2864 | 4.4 | C_20_H_36_N_2_O_2_ |

ID = Base Peak Cromathogram in positive ionization mode.
